# The moderating effect of psychological distress in the association between temperaments and dark future among young adults

**DOI:** 10.1186/s12888-023-05486-1

**Published:** 2024-01-03

**Authors:** Emmanuelle Awad, Diana Malaeb, Feten Fekih-Romdhane, Souheil Hallit, Sahar Obeid

**Affiliations:** 1 School of Arts and Sciences, Social and Education Sciences Department, Lebanese American University, Byblos, Lebanon; 2 College of Pharmacy, Gulf Medical University, Ajman, United Arab Emirates; 3 The Tunisian Center of Early Intervention in Psychosis, Department of Psychiatry “Ibn Omrane”, Razi hospital, Manouba, 2010 Tunisia; 4 Faculty of Medicine of Tunis, Tunis El Manar University, Tunis, Tunisia; 5 School of Medicine and Medical Sciences, Holy Spirit University of Kaslik, P.O. Box 446, Jounieh, Lebanon; 6 Applied Science Research Center, Applied Science Private University, Amman, Jordan

**Keywords:** Psychological distress, Temperament, Future anxiety, Lebanon

## Abstract

**Objective:**

The aim of the current study is to evaluate the moderating effect of psychological distress variables, depression, anxiety and stress on the relationship between affective temperaments and future anxiety, assessed with the Dark Future scale.

**Methods:**

Lebanese adults from all districts/governorates of Lebanon participated in this cross-sectional study. The data was collected through a questionnaire including: a section about sociodemographic characteristics, the Dark Future scale (DFS), the Depression Anxiety Stress Scale (DASS-8) and the Affective Temperament Scale (TEMPS-M).

**Results:**

The interaction irritable temperament by psychological distress (*p* = .007) was significantly associated with dark future; at low levels of psychological distress, more irritable temperament (Beta = 0.16) was significantly associated with more dark future. The interaction anxious temperament by psychological distress (*p* = .010) was significantly associated with dark future; at low (Beta = 0.34), moderate (Beta = 0.25) and high (Beta = 0.15) levels of psychological distress, more anxious temperament was significantly associated with more dark future.

**Conclusion:**

The nature of the associations among depression, anxiety and stress, specific temperaments and anxiety towards the future in a sample of Lebanese individuals was clarified. This is especially significant as Lebanese people live in circumstances that promote psychological distress and future anxiety such as dramatic economic and political crises, instability and lack of security in different aspects of life.

## Introduction

Temperament can be defined as a genetic base for tendencies that differentiate individuals from one another, and predisposes individuals towards certain reactivity patterns [1]. It can be considered as the rigid core of personality involved in emotion and self-regulation [2]. Different affective temperaments, including different temperament traits, can be identified: depressive, cyclothymic, hyperthymic, irritable and anxious, according to extensive research by Akiskal and colleagues [3]. These types were initially concluded from observations of psychopathology patients and linked to different mood disorders [4]. The depressive temperament is characterized by low affect, self-esteem and energy as well as the tendency for social isolation [5]. The cyclothymic temperament includes rapid shifts and instability in emotions, self-esteem and energy, cycling from high to low [6]. As for the hyperthymic temperament, impulsivity, hyperactivity, high self-esteem and intense positive mood are prominent elements [7]. Individuals with irritable temperament are likely to exhibit quickness to anger, tendency to be verbally and/or physically violent and skepticism [5]. In the anxious temperament, individuals have the tendency to show excessive worrying and exaggerated fear of something bad happening [5].

Fear and worry are closely associated with future anxiety, the tendency to regard the future in a pessimistic way and to expect the occurrence of negative events [8]. Future anxiety has been previously presented as a personality feature that promotes dark anticipation [9]. Zaleski developed a scale to measure the degree of future anxiety [9], to which a short version was developed called the Dark Future scale (DFS) [8]. The depressive temperament, associated with depression, describes negative views of the self but also of the future [10]. Similarly, the anxious temperament, associated with anxiety, illustrates overpowering expectations of threatening events in the future [10]. Furthermore, the original Future Anxiety scale had a positive relationship with both the cyclothymic and irritable temperaments [10].

Depression can be considered the clinical psychopathology associated with the anxious temperament or personality characteristic [11]. Temperaments that are negative in affect are closely related to depression and stress [12]. Cyclothymic and anxious temperament had a positive relationship with stress as well [13]. Cyclothymic, hyperthymic and depressive temperaments were considered to be significant affective elements in mood disorder including depression [14]. As for the anxious temperament, it was linked with the development of anxiety disorders in the past [15]. Temperament that involves excessive fear and worry was proven to be a risk factor for the emergence of anxiety disorders [16]. Akiskal proposed that anxiety is a type of anxious temperament, and that it can also be a basic risk factor for depression [17]. Depression or Major Depressive Disorder is a clinical disorder that involves symptoms such as low mood, apathy, increased or decreased appetite and sleep disturbances lasting at least two weeks [18]. Meanwhile, a depressive temperament is a biological enduring pattern of traits and responses, characterized by symptoms shared with depression, but also serves as a basis for an individual’s personality [14]. Overall, individuals exhibiting high levels of the depressive, cyclothymic, irritable and anxious temperaments showed higher psychological distress [19].

It can be inferred that temperaments, future anxiety and psychological distress are interrelated factors. Rationally, the relationship between the anxious temperament and future anxiety is rather reasonable as this temperament involves excessive fear of or worry about anticipated events, which then can cause a higher level of psychological distress, specifically anxiety. In fact, a fixed anxious mindset was found to be a predictor of psychological distress in the future [20]. However, the association between other affective temperaments, dark future, and depression, anxiety and stress are unclear. It can be hypothesized that the degree of psychological distress experienced can determine the effect of an inborn affective temperament on the probability of experiencing fear of the future and expecting negative events.

In Zaleski’s initial development of the DFS, it was stated that the need for the measurement stemmed from the worsening of the social, economic and political states worldwide, which subsequently affect psychological health critically [8]. Lebanon is a Middle Eastern country that has suffered major socio-economic devastation in the short span of four years. As a result, assessing the interaction of psychological factors using a tool designed towards significant negative shifts in general states of living can offer insight on the effect it has on the Lebanese population. The aim of the current study is to evaluate the moderating effect of psychological distress variables, depression, anxiety and stress on the relationship between affective temperaments and future anxiety, assessed with the DFS.

## Methods

### Study design

Between, Lebanese adults from all districts/governorates of Lebanon (Mount-Lebanon, Beirut, South, North, Nabatieh, Akkar and Bekaa) participated in this cross-sectional study. Using a snowball sampling approach, a survey was created on Google Forms and circulated across messaging applications and social media networks. Indeed, participants were first invited to complete the questionnaire which link was initially distributed via social media applications such as ‘WhatsApp’, ‘Instagram’ and ‘Facebook’, and then asked to share it with their acquaintances, friends and/or family members. First recruited participants comprise acquaintances of the present researchers, to whom they are connected on their social media accounts. In other words, the former would have visited the Google Forms link shared on the said online platforms, originally on the researchers’ profile, then completed the study’s questionnaire and followed with the Snowball Sampling method for further data collection. Before the survey was launched, a pilot test involving 15 people was performed to examine the survey’s feasibility and readability.

The study’s sample consisted of 684 Lebanese adults; all participants above the age of 18 were eligible to participate, and were given the assurance that it was completely voluntary, anonymous, and confidential. Prior to enrollment and informed consent, respondents were given information about the study’s goals and instructions. Excluded were those who refused to fill out the survey. No credits were awarded for participating.

### Minimal sample size calculation

For a 5% alpha error, a power of 80%, and 13 factors to be included in the linear regression models, a minimum of 395 participants would be needed, according to calculations by the G-power software.

### Questionnaire

The survey was written in Arabic, Lebanon’s official language, and consisted of three sections. The first section included a consent form, which verified the participants’ willingness to take the survey at their own discretion. A basic description of the study and instructions were also included in the first part. The second portion contained questions that assessed the participants’ socio-demographic information (age, sex, education level and marital status). The survey also asked participants if they had a personal history of mental illnesses. The Household Crowding Index (HCI) was computed by dividing the total number of residents by the total number of rooms in the household [21]. The socioeconomic status (SES) of the family is reflected by this measure, hence a higher HCI denotes a lower SES. Regarding financial burden, respondents were asked to answer the question “How much pressure do you feel with regard to your personal financial situation in general?” on a scale from 1 to 10, with 10 referring to overwhelming pressure. The third part of the survey comprised the following scales:

*Dark Future Scale (DFS).* The DFS was used to measure concern and anxiety toward the future [8]. The DFS is a 5-item scale (e.g., “I am afraid that the problems which trouble me now will continue for a long time”), scored on a 7-point Likert-type scale, varying from 0 (decidedly false) to 6 (decidedly true). A high score on the DFS reflects greater levels of future anxiety (Cronbach’s alpha = 0.89).

*Depression Anxiety Stress Scale (DASS-8).* The DASS-8, a shortened version of the DASS-21, consists of eight items divided into three subscales: depression (3 items), anxiety (3 items), and stress (2 items) [22]. Responses to the items are scored on a 4-point scale, ranging from 0 (did not apply to me at all) to 3 (applied to me very much or most of the time). The overall score of the DASS-8 ranges from 0 to 24, whereas the subscale scores range from 0 to 9, 0 to 9, and 0 to 6, respectively. Higher scores indicate a higher level of symptom affirmation (Cronbach’s alpha = 0.90).

*Affective Temperament Scale (TEMPS-M).* Affective temperament traits were assessed by the brief version of the Temperament Evaluation of Memphis, Pisa, Paris, and San Diego (TEMPS-M) [23], validated in Lebanon [24]. This scale is composed of 35 self-rating items that can be assigned to 5 subscales: depressive, cyclothymic, hyperthymic, irritable, and anxious. All responses are provided on 6-point Likert scales ranging from 1 (not at all) to 5 (very much). Each subscale score ranges from 5 to 35, with higher scores reflecting higher expressions of the respective temperament. The Cronbach’s alpha values were as follows: depressive 0.87, cyclothymic 0.89, hyperthymic 0.85, irritable 0.85, and anxious 0.87.

### Statistical analysis

The SPSS software v.25 was used for the statistical analysis. The psychological distress score was considered normally distributed since the skewness ( = − 0.458) and kurtosis ( = − 0.729) values varied between − 1 and + 1 [25]. The Student t was used to compare two means and the Pearson test was used to correlate two continuous variables. The moderation analysis was conducted using PROCESS MACRO (an SPSS add-on) v3.4 model 1 [26], taking each temperament as an independent variable, psychological distress as the moderator and dark future as the dependent variable. Results adjusted over all variables that showed a *p* < .25 in the bivariate analysis. *P* < .05 was deemed statistically significant.

## Results

### Sociodemographic and other characteristics of the sample

Six hundred eighty-four individuals participated in this study, with a mean age of 21.74 ± 4.30 years and 65.6% females. Other descriptive statistics of the sample can be found in Table 1.

**Table 1:** Sociodemographic and other characteristics of the sample (N = 684)

Variable	N (%)
Sex	
Male	235 (34.4%)
Female	449 (65.6%)
Marital status	
Single	643 (94.0%)
Married	41 (6.0%)
Education level	
Secondary or less	13 (1.9%)
University	671 (98.1%)
Region of living	
Urban	330 (48.2%)
Rural	354 (51.8%)
	**Mean ± SD**
Age (years)	21.74 ± 4.30
Household crowding index (persons/room)	1.44 ± 0.76
Financial satisfaction	5.59 ± 2.60

### Bivariate analysis of factors associated with dark future

The results of the bivariate analysis of factors associated with dark future are summarized in Tables 2 and 3. The results showed that females had higher mean dark future scores than males. Moreover, higher depressive, cyclothymic, irritable and anxious temperaments, higher psychological distress, household crowding index and financial burden were significantly associated with higher dark future scores, whereas older age was significantly associated with lower dark future scores.

**Table 2:** Bivariate analysis of factors associated with dark future scores

Variable	Mean ± SD	*p*
Sex		**0.001**
Male	16.49 ± 8.03	
Female	18.67 ± 7.71	
Marital status		0.857
Single	17.94 ± 7.85	
Married	17.71 ± 8.44	
Education level		0.271
Secondary or less	15.54 ± 10.21	
University	17.97 ± 7.83	
Region of living		0.919
Urban	17.89 ± 7.67	
Rural	17.95 ± 8.08	

Numbers in bold indicate significant *p* values

**Table 3:** Correlations of continuous variables with dark future

	1	2	3	4	5	6	7	8	9	10
1. Dark future	1									
2. Depressive temperament	0.48***	1								
3. Cyclothymic temperament	0.51***	0.71***	1							
4. Hyperthymic temperament	0.004	− 0.08*	0.03	1						
5. Irritable temperament	0.20***	0.55***	0.48***	0.08*	1					
6. Anxious temperament	0.40***	0.57***	0.59***	0.06	0.43***	1				
7. Psychological distress	0.42***	0.68***	0.67***	− 0.11**	0.47***	0.64***	1			
8. Age	− 0.09*	− 0.07	− 0.12**	0.08*	− 0.04	− 0.09*	− 0.08*	1		
9. Household crowding index	0.09*	0.04	0.03	− 0.01	− 0.02	0.06	0.06	− 0.12**	1	
10. Financial satisfaction	0.14***	0.10*	0.07	0.02	0.04	0.05	0.09*	0.01	0.08*	1

*p < .05; **p < .01; ***p < .001

### Moderation analysis with dark future taken as the dependent variable

The details of the moderation analysis of psychological distress taken as a moderator in the association between each temperament and dark future, are summarized in Table 4. The results were adjusted over age, gender, household crowding index and financial satisfaction. The interaction irritable temperament by psychological distress (*p* = .007) was significantly associated with dark future (Table 4, Model 4); at low levels of psychological distress, more irritable temperament (Beta = 0.16) was significantly associated with more dark future (Table 5, Model 1).

**Table 4:** Moderation analysis taking each temperament as an independent variable, psychological distress as a moderator and dark future as the dependent variable

Moderator	Beta	*T*	*P*	95% CI
**Model 1: Depressive temperament as an independent variable**				
Depressive temperament	0.54	6.47	**< 0.001**	0.38; 0.71
Psychological distress	0.34	2.84	**0.005**	0.11; 0.58
Interaction depressive temperament by psychological distress	− 0.01	-1.53	0.127	− 0.02; 0.002
**Model 2: Cyclothymic temperament as an independent variable**				
Cyclothymic temperament	0.48	6.83	**< 0.001**	0.34; 0.62
Psychological distress	0.23	1.66	0.098	− 0.04; 0.51
Interaction cyclothymic temperament by psychological distress	− 0.003	− 0.54	0.590	− 0.01; 0.01
**Model 3: Hyperthymic temperament as an independent variable**				
Hyperthymic temperament	0.19	2.19	**0.029**	0.02; 0.37
Psychological distress	0.79	4.17	**< 0.001**	0.42; 1.17
Interaction hyperthymic temperament by psychological distress	− 0.01	-1.53	0.126	− 0.03; 0.003
**Model 4: Irritable temperament as an independent variable**				
Irritable temperament	0.23	2.55	**0.011**	0.05; 0.41
Psychological distress	0.77	6.68	**< 0.001**	0.54; 0.99
Interaction irritable temperament by psychological distress	− 0.02	-2.70	**0.007**	− 0.03; − 0.01*
**Model 5: Anxious temperament as an independent variable**				
Anxious temperament	0.40	4.84	**< 0.001**	0.24; 0.57
Psychological distress	0.64	5.07	**< 0.001**	0.39; 0.89
Interaction anxious temperament by psychological distress	− 0.02	-2.60	**0.010**	− 0.03; − 0.004*

*indicates significant moderation; numbers in bold indicate significant *p* values; results adjusted over age, gender, household crowding index and financial satisfaction

**Table 5:** Conditional effects of the focal predictor (each temperament) at values of the moderator (psychological distress)

Psychological distress	Beta	*T*	*P*	95% CI
**Model 1: Irritable temperament as the independent variable**.				
Low (= 3.88)	0.16	2.29	**0.022**	0.02; 0.30
Moderate (= 10.09)	0.05	1.02	0.308	− 0.05; 0.15
High (= 16.29)	− 0.06	-1.00	0.320	− 0.17; 0.06
**Model 2: Anxious temperament as the independent variable.**				
Low (= 3.88)	0.34	5.18	**< 0.001**	0.21; 0.47
Moderate (= 10.09)	0.25	4.92	**< 0.001**	0.15; 0.35
High (= 16.29)	0.15	2.65	**0.008**	0.04; 0.27

Numbers in bold indicate significant *p* values

The interaction anxious temperament by psychological distress (*p* = .010) was significantly associated with dark future (Table 4, Model 5); at low (Beta = 0.34), moderate (Beta = 0.25) and high (Beta = 0.15) levels of psychological distress, more anxious temperament was significantly associated with more dark future (Table 5, Model 2).

## Discussion

This study is amongst the first to investigate dark future in a sample of Arab and Lebanese population from the Middle East, its association with affective temperament traits, and moderating effects of distress in this association. Findings showed that psychological distress acted as a moderator in the relationship between two affective temperaments (i.e., irritable and anxious) and future anxiety. According to the bivariate analysis, sex was significantly associated with dark future. Although the sample was comprised of a majority of females (65.6%), congruent findings have been observed in the relationship between gender and future anxiety in different studies and populations. For instance, a previous Italian study found that the majority of the sample was also composed of women, and women scored higher on the DFS in comparison with men [27]. A study performed in a sample of gender proportionate Polish adults (50% females) showed that females exhibited higher future anxiety than males [8]. Another study revealed that mothers having a child with developmental disabilities displayed much more worries than fathers about their own future and that of their child [28]. Overall, women exhibit far more anxiety levels than men across their lifespan, and have a notably higher prevalence of anxiety disorders [29]. Given that dark future relates to excessive anxiety towards the future, it seems consistent that female sex shows a significant association with DFS scores.

The first assumption in this study was that experiencing psychological distress might impact the association between affective temperaments and exhibiting fear of the future. To begin with, previous research hypothesized that affective temperaments exist on a spectrum ranging from adaptive to pathological behaviors, potentially contributing to states of psychological distress [30]. Meanwhile, the nature of all the associations mentioned initially above were unclear. Therefore, the hypothesis was tested by conducting a moderation analysis, with psychological distress as a moderator between temperament and dark future.

The literature offers a very limited number of studies discussing temperament and anxiety related to the future, focusing more on anxiety disorders [31]. Given the current state of the world, the circumstances causing uncertainty can lead to worrying or fearing future events, especially among Lebanese people. Affective temperaments can influence individuals’ anxiety towards the future [32]. Previous research has confirmed that feel uncertain and helpless regarding the future can be predicted by temperament [33]. In the measure developed by Akiskal and colleagues, which is used in this study, an anxious temperament consists of fear and worry regarding others’ safety [5]. Similarly, the DFS used in this study assesses individuals’ degree of worry about negative events happening in the future in general, including natural disasters and war [8].

One finding of this study was that psychological distress moderated the relationship between irritable temperament and dark future. Another finding was that psychological distress moderated the relationship between anxious temperament and dark future. These results indicate that the relationship between certain temperaments, irritable, anxious, and dark future becomes more significant in individuals experiencing high depression, anxiety and stress. Generally, the dysregulation of affective temperamental patterns was connected to psychological distress [30]. Irritable temperament had a positive correlation with depression [34], with irritability predicting depression [35]. In contrast, another study [12] found that all affective temperaments, except irritable temperament, predicted depression, further showing conflict with past findings. Furthermore, irritable temperament was connected to more depressive episodes and more intense depressive symptoms [36], but not with the emergence of depression as a psychological disorder. This consolidates that the relationship between irritable temperament and psychological distress is not entirely explained through direct correlation. Higher anxiety was associated with the irritable temperament, with anxiety accurately predicting an irritable temperament [37]. Akiskal and his colleagues’ original description of the irritable temperament included intense anger outbursts and potential violence, with no mention of patterns of anxiety about the future [5]. However, previous research found that the presence of irritable temperament is linked with anxiety and can predict the intensity of anxiety behaviors [38]. Also, irritability, a central element of the irritable temperament, is potentially maintaining factor for psychological distress, including anxiety [39]. It can be assumed that the effect of irritable temperament is more severe on anticipating dark future events to happen when there is psychological distress, especially anxiety, which is directly related to and overlapping with future anxiety [40]. Meanwhile, Akiskal argued that anxiety could be a type of anxious temperament that was linked to depression [17], as previously mentioned. Therefore, having an anxious temperament and future anxiety are somewhat different in their target, what anxiety is directed towards, although the central characteristic of both is anxiety, which might justify the indirect association.

### Limitations

First, no studies were conducted on psychological distress, temperament and dark future previously, which means that there are no prior results to contrast with the ones from this study. Second, the data was collected through a self-report measure in this investigation, potentially causing responder bias. Third, limited by the length of our survey, we used the shortest version of the DASS. Future research should consider using the full-length version of the scale (DASS-21; [41]). Finally, the participants were recruited through a snowball sampling technique that might decrease the generalizability of the study. On the other hand, participants came from all Lebanese governorates.

### Clinical implications

The current results offer important practical implications both locally and internationally. Most notably, the findings might offer help in reducing the impact of dark future in individuals with irritable and anxious temperaments when treating psychological distress. With many parts of the world struggling with instability and crises, the findings can be used in the clinical setting to assess important psychological variables such as distress, temperament and future anxiety following major devastating events such as the recent pandemic and economic collapse globally.

## Conclusion

The results suggest that psychological distress is a moderator for the relationship between irritable temperament and dark future. Psychological distress is also a moderator for the relationship between anxious temperament and dark future. The nature of the associations among depression, anxiety and stress, specific temperaments and anxiety towards the future in a sample of Lebanese individuals was clarified. The study offers valuable insight on how experiencing psychological distress can influence the link between an inborn affective temperament and anticipating negative events in the future.

## Data Availability

All data generated or analyzed during this study are not publicly available due the restrictions from the ethics committee, but are available upon a reasonable request from the corresponding author (SH).
